# MDCT Findings of Denim-Sandblasting-Induced Silicosis: a cross-sectional study

**DOI:** 10.1186/1476-069X-9-17

**Published:** 2010-04-17

**Authors:** Cihan Akgul Ozmen, Hasan Nazaroglu, Tekin Yildiz, Aylin Hasanefendioglu Bayrak, Senem Senturk, Gungor Ates, Levent Akyildiz

**Affiliations:** 1 Dicle University School of Medicine Department of Radiology 21280, Diyarbakir, Turkey; 2 Dicle University School of Medicine Department of Chest Diseases and Tuberculosis 21280, Diyarbakir, Turkey

## Abstract

**Background:**

Denim sandblasting is as a novel cause of silicosis in Turkey, with reports of a recent increase in cases and fatal outcomes. We aimed to describe the radiological features of patients exposed to silica during denim sandblasting and define factors related to the development of silicosis.

**Methods:**

Sixty consecutive men with a history of exposure to silica during denim sandblasting were recruited. All CT examinations were performed using a 64-row multi-detector CT (MDCT). The nodules were qualitatively and semi-quantitatively analyzed by grading nodular profusion (NP) on CT images.

**Results:**

Silicosis was diagnosed radiologically in 73.3% of patients (44 of 60). The latency period (the time between initial exposure and radiological imaging) and duration of silica exposure was longer in patients diagnosed with silicosis than in those without silicosis (p < 0.05). Nodules were present in all cases with centrilobular type as the commonest (63.6%). All cases of silicosis were clinically classified as accelerated and 11.4% had progressive massive fibrosis (PMF). Mild NP lesions were the most prevalent in all six zones of the lung. The NP score was significantly correlated with the duration of silica exposure, the latency period, presence of PMF, and pleural thickening. Enlarged lymphadenopathy was present in 45.5% of patients.

**Conclusions:**

The duration of exposure and the latency period are important for development of silicosis in denim sandblasters. MDCT is a useful tool in detecting findings of silicosis in workers who has silica exposure.

## Background

Silicosis is an incurable lung disease caused by the inhalation of dust containing free crystalline silica. Extremely high exposures are associated with a short latency and rapid disease progression [1]. The condition is irreversible and progresses even when exposure stops. Silicosis is one of the oldest occupational diseases and kills thousands of people worldwide every year.

Silicosis occurs in two distinct clinical forms: acute silicosis, also known as silicoproteinosis, and classic or chronic silicosis. Acute silicosis occurs following exposure to a large quantity of silica, most frequently as a result of sandblasting. Classic or chronic silicosis is characterized by the development of nodular infiltrative lung disease [2] and occurs following long-term exposure (i.e., 10-20 years) to a low concentration of silica dust. Accelerated silicosis is similar to classic silicosis, but occurs following exposure to higher dust concentrations for a shorter period (as little as 5 years) [3]. Silicosis has two radiological forms: simple and complicated [2,3].

Denim sandblasting was recently identified as a novel cause of silicosis in Turkey, with reports of a recent increase in cases and fatal outcomes [4-8]. Denim sandblasting uses silica-containing sand as an abrasive on blue jean surfaces to produce a "worn-out" look. This exposure is more dangerous than others because it involves intense exposure for long periods under poor hygienic conditions and in the absence of ventilation systems or respiratory protection [5-8]. Acute silicosis can also occur in quartzite millers, tunnel workers, silica flour workers, and workers in the scouring powder industry [9,10].

Although chest radiography is the most convenient imaging technique for diagnosing and monitoring the progression of silicosis, it has some limitations in the assessment of pneumoconiosis [11]. Thin-section computed tomography (CT) has been shown to detect some cases of silicosis that chest X-ray did not, and the technique is able to better characterize changes to the lung [7,9,12,13]. Although the use of high-resolution CT (HRCT) and chest X-ray imaging in patients with silicosis has been well documented [10,14-17], there are few reports of multidetector CT (MDCT) imaging in patients with silicosis of denim sandblasting [4,5]. The aim of the present study was to describe the radiological features of patients exposed to silica during denim sandblasting and define the factors related to the development of this type of silicosis.

## Methods

### Patients

Between April 2008 and April 2009, 60 consecutive men admitted to the Chest Disease Department of Dicle University School of Medicine with a history of exposure to silica during denim sandblasting were recruited for the study.

The patients lived in rural areas of Diyarbakir and worked as denim sandblasters in various factories in Istanbul. They were admitted to the hospital after learning about the severity of the disease from our previously diagnosed patients. Patients with active tuberculosis or a history of the disease were not included in the study. Informed consent was obtained from all study participants. The diagnosis of silicosis was based on a history of exposure to silica-containing dust and radiological changes consistent with silicosis. Chest radiography and CT imaging were performed on all patients. Demographic and clinical characteristics, including age, exposure duration, smoking history, and latency period, were recorded. The latency period was defined as the time between initial exposure and radiological imaging. Cigarette consumption and occupational silica exposure were quantified in terms of the number of packs/year and number of months of exposure, respectively. Patients were divided into simple and complicated silicosis on the basis of CT findings. Similar to the literature [3], silicosis was described as simple when multiple small nodules appeared on the CT image and as complicated when PMF was detected. Patients were categorized into acute, chronic, or accelerated silicosis using clinical indicators. The chronic form was used for the silicosis after 10-20 years of exposure to low concentrations of silica dust. An accelerated form of silicosis was defined in those with silicosis that occurred within 4-10 years of heavier exposure [13,14].

### CT imaging

All CT examinations were performed using a 64-row multidetector CT system (Brilliance CT scanner, Philips Healthcare) and images from the lung apices to the lung bases were obtained during inspiration in the supine position. The acquisition parameters were: 120 kV; 300 mAs; detector collimation, 64 × 0.625 mm; and slice thickness, 3 mm. Contrast medium was not used. In addition, HRCT images of 1 mm thickness were reconstructed. All images were reviewed independently by two radiologists with experience in thoracic imaging who were unaware of the clinical data. If there was a disagreement between the readers, the CT scans were again reviewed by both readers and a final decision was reached by consensus.

The presence of lymphadenopathy and calcification were investigated in a mediastinal window. Calcifications were classified as central-, eccentric-, or eggshell-type. The lung parenchyma was evaluated using HRCT sections at - 600 window level (WL) and 1600 window width (WW). The sections were evaluated in the coronal, sagittal, and axial planes using a Philips workstation (Extended Brilliance Workspace, Philips Healthcare). Lung nodules were evaluated using 5-mm-thick maximum intensity projection (MIP) reconstructions.

Several radiological findings were recorded including: distribution of the nodules, interlobular and intralobular reticulation, parenchymal bands, ground-glass opacity, consolidation, atelectasis, progressive massive fibrosis (PMF) or masses, bronchiectasis, and emphysema. Pleural thickening, effusion, and calcification were also recorded.

Each lung was divided into upper (apex to carina), middle (carina to inferior pulmonary vein), and lower (inferior pulmonary vein to lung base) zones. The nodules were qualitatively and semi-quantitatively analyzed using a previously reported system for grading nodular profusion (NP) on CT images [5,18]. The grades were defined as follows: grade 0, no nodules present; grade I, a small number of nodules without vascular obliteration; grade II, several nodules with mild vascular obliteration; grade III, several nodules with moderate vascular obliteration; and grade IV, several nodules with severe vascular obliteration with or without coalescence (< 1.5 cm).

After grading (0-4) each of the six lung zones, an overall CT NP grade was calculated from the sum of the grades assigned to each lung zone. The presence of nodules was evaluated in two ways: an anteroposterior distribution in which the lung was divided into ventral, middle, and posterior thirds, and an axial distribution in which the lung was divided into central, middle, and peripheral thirds. The nodule type was labeled as centrilobular, perilymphatic, and randomized nodules according to the most prevalent type in a patient. The presence of a few nodules of different type was ignored. PMF was defined as the presence of an opacity or coalescence larger than 1.5 cm in diameter on the CT image [18]. The presence of emphysema was also investigated.

We investigated the correlation between the CT grade and time of exposure to silica, the latency period, age at admission to the hospital, smoking, presence of PMF, and pleural thickening.

### Statistical analysis

Results are expressed as the mean ± standard deviation or as a percentage. Between-group comparisons were performed using the Student's t-test for parametric values and the Mann- Whitney U-test, as appropriate. A p-value of < 0.05 was considered statistically significant. A receiver operating characteristic (ROC) curve analysis was performed to investigate the role of related parameters in the development of radiological silicosis. Spearman's rank correlation coefficient was calculated to evaluate the relationship between the duration of silica exposure, the latency period, the CT NP grade, and the presence of PMF.

## Results

### Patient characteristics

The mean age of the 60 patients was 26.0 ± 5.5 years (range, 17-43 years) and all of the participants were men. All patients worked as denim sandblasters and had a history of exposure to silica varying from 2-60 months (18.5 ± 18.4 months). The mean latency period was 7.3 ± 1.6 years (range, 5-13 years). Of the 60 patients, 58% had a history of smoking. The mean number of packs/year was 7.7 ± 6.7 years in 35 smokers.

**Figure 1:**
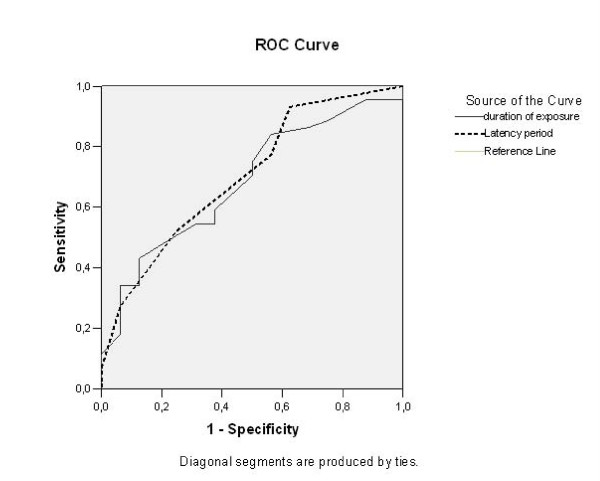
**The areas under the curve (AUC) for the duration of exposure to denim sandblasting (line) and the latency period (dashed line) were 0.703 and 0.683, respectively**.

Silicosis was diagnosed radiologically in 44 patients (73.3%). The latency period was 7.6 ± 1.5 years in patients with radiological findings of silicosis; this was significantly longer than for patients in whom silicosis was not detected (6.5 ± 1.4 years; p = 0.012). The duration of silica exposure was also longer in patients diagnosed with silicosis (21.4 ± 19.6 months) than in those without silicosis (10.5 ± 10.8 months; p = 0.031). The mean age of the two groups was similar. The percentage of smokers was higher in the silicosis patients (70.5%) than in those without silicosis (50%; p = 0.142). However, the mean number of packs/year did not differ between patients with or without silicosis (7.8 ± 7.0 versus 7.6 ± 5.6 years, respectively; p = 0.956). The ROC curve analysis of duration of exposure to denim sandblasting and the latency period for development of radiological silicosis showed that the areas under the curve (AUC) were 0.703 and 0.683, respectively (Figure 1).

### Nodules and PMF

All cases of silicosis were clinically classified as accelerated (n = 44). Of the 44 patients, five (11.4%) had PMF indicating complicated silicosis, and the remaining 39 men (88.6%) had simple silicosis. PMF was detected in the upper lobes and was located in the posterior region of the lungs in all five cases. In two of those cases, the middle zone of the anteroposterior axis was also involved.

Grade 1 lesions were the most prevalent in all six zones of the lung. NP was more severe in the upper zones than in the lower zones. The NP grades for each of the six zones are shown in Table 1.

**Table 1:** Grade distribution in each of the six lung zones

	Grade 1		Grade 2		Grade 3		Grade 4		Total	
**Zone**	R %(n)	L %(n)	R %(n)	L %(n)	R%(n)	L%(n)	R%(n)	L%(n)	R %(n)	L %(n)

**Upper**	48 (21)	41(18)	18 (8)	21 (9)	5 (2)	7 (3)	16 (7)	11(5)	86.4(38)	79.5(35)

**Middle**	50 (22)	39 (17)	25 (11)	27 (12)	7(3)	2 (1)	0 (0)	2 (1)	81.8(36)	70.5(31)

**Lower**	52 (23)	41(18)	30 (13)	27 (12)	0(0)	2 (1)	0(0)	2 (1)	81.8(36)	72.7(32)

We observed diffuse distribution of nodules in the anteroposterior axis. Nodules were found in all lung regions (anterior, middle, and posterior) in 30 patients, in two regions in eight patients (anterior and middle and anterior and posterior in two patients each, and middle and posterior in four patients), and six patients had nodules in one lung region (anterior in one patient and posterior in five patients). The posterior region of the lung was most commonly affected, and nodules were found there in 41 patients.

We found nodules in all lung regions in 22 patients, in two regions in 11 patients (all mid-peripheral), and in a single zone in 11 patients (all peripheral) of the central- peripheral axis. The most common region of involvement was the peripheral one (44 patients) in the central- peripheral axis.

The most common nodule type was centrilobular; it was detected in 28 patients (63.6%). Perilymphatic nodules were found in seven patients (15.9%), and randomized nodules were found in six patients (13.6%).

### Lymhadenopathy

Enlarged lymphadenopathy was present in 20 patients (45.5%). Calcification was observed in four cases: two were central and eccentric and two were central. We detected no eggshell calcification.

### NP score and correlations

The mean NP score was 7.6 ± 5.1 (range, 1-18). The NP score upon CT in the 44 patients with silicosis was significantly correlated with the duration of silica exposure (r = 505, p < 0.0001), the latency period (r = 405, p = 0.006), presence of PMF (r = 542, p < 0.0001), and pleural thickening (r = 519, p < 0.0001). However, the correlation between NP score and age or smoking was not significant.

### Other radiological findings

Other radiological findings detected upon CT were: ground glass opacity in 18.2%, consolidation in 2.3%, traction bronchiectasis in 6.8%, centrilobular emphysema in 27.3%, paracicatricial emphysema in 18.2%, reticular opacity in 22.7%, interlobular septal thickening in 31.8%, intralobular reticulation in 4.5%, and parenchymal bands in 38.6% of the 44 patients. In addition, central bronchiectasis was detected in 13.6% of the patients. Pleural thickening was present in 15 patients (34.1%), which was plaque-like in 12 cases, nodular in two cases, and plaque-like and nodular in one case. Pleural effusion was not detected.

## Discussion

Silicosis is one of the oldest occupational diseases and well-documented. Although chest X-ray is widely used to diagnosis and follow up the disease, there are several reports of CT detection of chronic silicosis [15,17-19]. Denim sandblasting is a novel cause of silicosis that has recently been reported in Turkey; thus, there are few reports in the literature on CT detection of accelerated [4] and acute silicosis [10,14] caused by denim sandblasting. Most of these studies used HRCT or spiral CT, whereas diagnosis using MDCT has been reported in only five cases [4,5]. In the present study we used 64-row MDCT to detect accelerated silicosis.

Silicosis was described radiologically in 73.3% of our patients (44 of 60 patients). Our detection rate is higher than that reported by Akgun et al. [16] who detected silicosis in 53% of patients (77 of 145 patients) using chest X-ray. The patients in their study were of a similar age, but had a longer latency period and duration of exposure to denim sandblasting than our patients. This discrepancy in results may be explained by two factors. First, CT scans are superior to chest X-rays in the early detection of the initial phases of silicosis [17] and CT has been reported to detect changes in nodular coalescence in silicosis earlier than chest X-ray [20]. Therefore, the higher detection rate of silicosis in our study may be explained by the use of MDCT versus chest X-ray. The second explanation may be the difference in concentration of silica dust inhaled by the workers in the two studies.

In the present study, the latency period and duration of exposure were longer in patients diagnosed with silicosis. However, no significant differences in age, percent of smokers, or number of packs/year were found between men with or without silicosis. Duration of exposure to silica and the latency period had high areas under curve in ROC analysis and therefore they were significant factors with for the development of radiological findings of silicosis. Similarly, Akgun et al. [16] reported that patients with silicosis had a longer latency period and longer exposure than patients who did not have silicosis, and they found no significant difference in age or smoking history between the groups.

### Nodules

**Figure 2:**
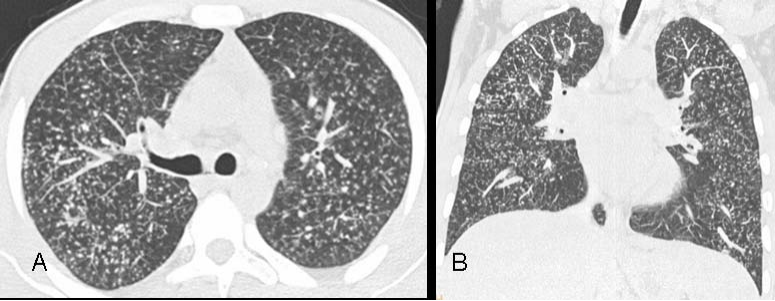
**a, b - Diffuse centrilobular nodules on 5-mm-thick reconstructed axial (a) and coronal maximum intensity projection (MIP) images (b) in a 24-year-old man who worked as a denim sandblaster for 48 months**.

Pulmonary nodules in the lung parenchyma are usually present in silicosis and typically located in the upper lobes [9]. They are common in both acute and accelerated silicosis and are present in 85-94% of the cases [4,10]. Nodules are also present in 94-100% of chronic silicosis cases [17,21,22]. We detected nodules in every case of silicosis in the present study. The most common type was centrilobular and they were found in 63.6% of the patients (Figure 2). Similarly, Alper et al. [4] reported detecting centrilobular nodules in 60% of their patients. In addition, the predominance of centrilobular nodules has been reported in patients with acute silicosis [10].

**Figure 3:**
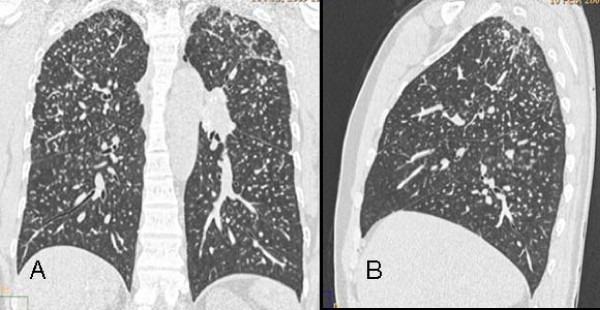
**a, b - Coronal (a) and sagittal (b) reconstructed computed tomography images showing the predominance of nodules located in the upper lobes and peripheral regions of the lungs in a 29-year-old man who worked as a denim sandblaster for 20 months**.

Mild nodular profusion was common and grade 1 nodular lesions were the most prevalent in the six zones of lung. The most severe nodular profusions were located in the upper zones (Figure 3). Using the same grading system as ours, Alper et al. [4] reported a middle to upper zone predominance in milder cases (grades I and II) and middle to lower zone predominance in severe cases (grades III and IV) of nodular profusion. These findings are in contrast to ours and those previously reported in the literature [13].

We observed a diffuse distribution of nodules in the anteroposterior axis in 30 patients. The posterior region was involved in 41 patients, and nodules were distributed in the middle to peripheral region of the central- peripheral axis. A peripheral distribution was the most common and appeared in all patients with silicosis. These findings are consistent with other reports in the literature [4,11,21].

### PMF

PMF has a prevalence of 75% in chronic silicosis [17], and is also present in accelerated silicosis. Alper et al. [4] detected PMF in 32% of their patients with accelerated silicosis; however, we observed PMF in only 11.4% (five of 44) of our patients with accelerated silicosis. The shorter duration of exposure experienced by our patients may account for this difference (21.4 ± 19.6 versus 30.4 ± 19.0 months).

**Figure 4:**
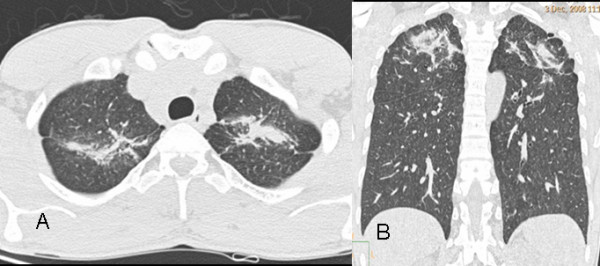
**a, b - PMF and bands leading to parenchymal distortion in adjacent regions in both upper lobes of the lung are apparent on axial (a) and coronal (b) CT sections of a 27-year-old man who worked as a denim sandblaster for 48 months**.

Of our 44 patients with silicosis, five (11.4%) had complicated silicosis, as defined by the presence of PMF, and the remaining 39 men (88.6%) had simple silicosis. Our findings agree with those in the literature suggesting that PMF is most commonly located in the posterior region of the upper lobes of the lungs in patients with denim sandblasting-induced silicosis [4,15] (Figure 4).

### Lymph node enlargement

**Figure 5:**
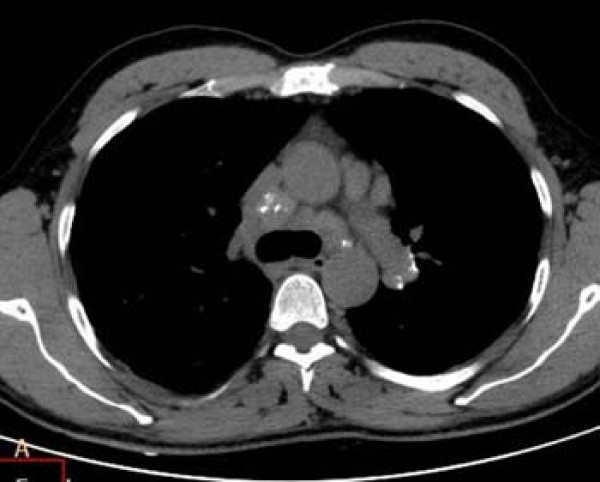
**CT images of a 29-year-old man who worked as a denim sandblaster for 20 months**. Paratracheal and left side hilar lymph nodes with central and eccentric calcification are shown.

Lymph node enlargement was detected in 63.6% of the patients with chronic silicosis in a study using HRCT imaging [17]. Alper et al. [4] reported lymph node enlargement in half of their cases with accelerated silicosis; furthermore, they reported calcification in six (12%) patients (four eccentric and two central). Enlarged lymphadenopathy was present in 20 (45.5%) of the patients in the present study (Figure 5). Of those, four (9.1%) were calcified, two cases were central, and two were central and eccentric. In a previous study, calcified lymph nodes were reported in 11 of 13 (85%) patients with acute silicosis, and were located predominantly in the hilar regions and showed diffuse nodal calcification [10]. These patients were exposed to silica longer than the patients in our study and the latency period was not available; thus, it is possible that silicosis was more severe in these patients (acute type) than in our patients or those described by Alper et al. [4].

### Pleural thickening

**Figure 6:**
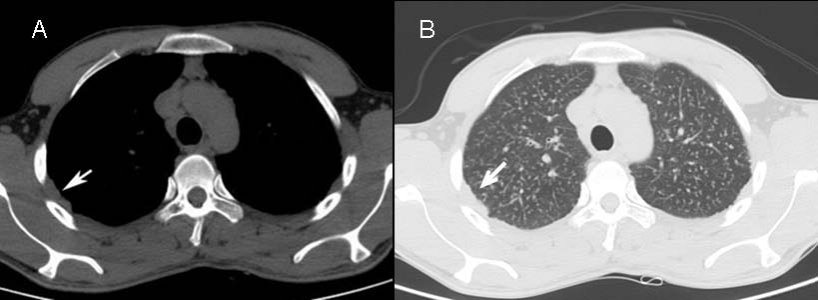
**The right-sided pleural thickening in a 23-year-old man who worked as a denim sandblaster for 36 months**. (a: mediastinal window, b: lung window).

In previous studies, pleural thickening was reported in 31.8-58% of patients with chronic silicosis on CT images [17,19], and in 38% of patients with accelerated silicosis on CT [4]. Minimal pleural thickening or pleural effusion had been reported in 85% of 13 patients with acute silicosis [10]. We found pleural thickening in 34.1% of our patients (Figure 6). Similar to findings in other patients with accelerated silicosis [4], pleural thickening was plaque-like in 12 cases, nodular in two cases, and plaque-like and nodular in one case. Pleural thickening is reportedly more common in complicated silicosis than in simple silicosis in both the chronic [19] and accelerated [4] forms of the disease. In our study, four of five cases (80%) with complicated silicosis also showed pleural thickening. All cases in our study has pleural thickening less than 5 cm length and can be classified as segmented pleural thickening according the classification used by Arakawa et al [19]. The presence of no case with diffuse pleural thickening (longer than 5 cm) may be due to involvement of no cases with chronic silicosis in our study.

### NP score and correlations

The NP score on CT images in patients with radiological silicosis was significantly correlated with the duration of silica exposure, the latency period, the presence of PMF, and pleural thickening. However, no significant correlation was found between the NP score and age or smoking in the present study. Alper et al. [4] also reported a significant correlation between nodular CT grade and duration of silica exposure, latency period, the presence of PMF, and pleural thickening.

### Other radiological findings

Emphysematous changes may be present in patients with chronic silicosis [9]. centrilobular emphysema was detected in 27.3% of our patients, and paracicatricial emphysema was found in 18.2%. In a previous study, emphysema was reported in 27 cases (61.4%): 21 were scar-related emphysema and six were panacinar emphysema. Twenty-four of those cases also showed PMF on HRCT in patients with chronic silicosis [17]. Alper et al. [4] reported emphysematous changes in 18% of their cases. However, no emphysema was reported in silicoproteinosis [10]. The higher rate of emphysematous changes detected in our study compared to those described by Alper et al. [4] may be explained by the exclusion of heavy smokers in their study.

Ground-glass opacities are characteristic of acute silicosis [3,9]. However, ground glass opacities were present in 18.2% of our patients, all of whom were clinically diagnosed with accelerated silicosis. Similarly, Alper et al. [4] reported ground glass opacities in 12% of their accelerated silicosis patients. Therefore, ground-glass appearance may also been found in patients with accelerated silicosis.

**Figure 7:**
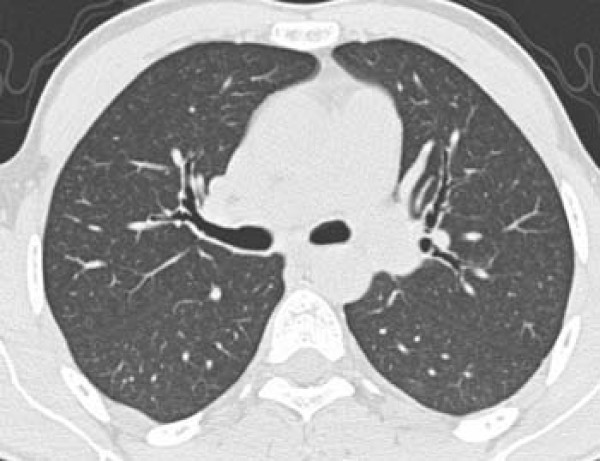
**Mild dilatation in the bronchus of the upper lobes and thickening of the bronchial wall is apparent in a 27-year-old man who worked as a denim sandblaster for 20 months**.

Centrally located mild bronchiectasis was detected in 13.6% of our patients (Figure 7) Traction bronchiectasis was present in 6.8% of the patients in our study. This is in agreement with Alper et al. [4], who reported traction bronchiectasis in 8% of their patients with accelerated silicosis. In addition, one study detected bronchiectasis in 64.7% of patients with chronic silicosis [22]. Minimal architectural distortion and traction bronchiectasis has been reported in four (31%) patients with acute silicosis [10].

Other radiological findings detected upon CT were consolidation, reticular opacity in interlobular and intralobular reticulation, and parenchymal bands. Consolidation is reportedly frequent in acute, but not in accelerated, silicosis [3,10] which was the case in our study.

Our study has several limitations. First, our patient group may not represent the entire population of denim sandblasters. Secondly, information on the exact type of silica and the concentration to which patients were exposed was not available. Although we used well-defined criteria for diagnosis, the study lacks pathological confirmation. However, we believe that we met our aim to define the radiological features of silicosis in sandblasters. Although all patients exposed to silica dust were included all were classified as "accelerated" so in fact this paper really describes the findings in patients clinically diagnosed with accelerated silicosis. The reason the current population is comprised entirely of accelerated silicosis, may be a matter of survival selection, that is, the acute silicoproteinosis cases did not survive to be included in the study.

## Conclusions

The duration of exposure and the latency period are important factors in the development of radiological silicosis in men who are exposed to silica during denim sandblasting. MDCT findings of accelerated silicosis consist of centrilobular nodules in the lung parenchyma, typically in the posterior portion of the lungs with peripheral distribution. PMF is not as common in accelerated silicosis as it is in chronic silicosis. Enlarged lymphadenopathy is common in patients with accelerated silicosis. However, calcification is rare. Plaque-like pleural thickening is the most common pleural finding, and was present in half of patients in this study. The NP score on CT images is a marker of advanced disease. Ground-glass opacities, although rare, may also be observed in accelerated silicosis.

## List of Abbreviations

CT: computed tomography; MDCT: multi-detector CT; PMF: progressive massive fibrosis; NP: nodular profusion; HRCT: high-resolution CT; MIP: maximum intensity projection; WL: window level; ROC: receiver operating characteristic; AUC: areas under the curve.

## Competing interests

The authors declare that they have no competing interests.

## Authors' contributions

CAO has contributed conception and design of the study, analysis and interpretation of data, drafting the manuscript for important intellectual content; and has given final approval of the version to be published. HN has given final approval of the version to be published. TY has contributed the conception and design, acquisition of data. AHB and SS has also involved in acquisition of data and interpretation of data. GA has contributions to conception and design, acquisition of data and involved in drafting the manuscript. LA was involved in acquisition of data, analysis and interpretation of data. All authors have read and approved the final manuscript.
